# Removal of Chromium(VI) from Aqueous Solutions Using Fe_3_O_4_ Magnetic Polymer Microspheres Functionalized with Amino Groups

**DOI:** 10.3390/ma8125461

**Published:** 2015-12-03

**Authors:** Kai Wang, Guangming Qiu, Hongyu Cao, Ruifa Jin

**Affiliations:** 1 Inner Mongolia Key Laboratory of Photoelectric Functional Materials, College of Chemistry and Chemical Engineering, Chifeng University, Chifeng 024000, China; hongyucao20151130@gmail.com (H.C.); ruifajin20151130@gmail.com (R.J.); 2 College of Energy and Power Engineering, Inner Mongolia University of Technology, Huhhot 010051, China

**Keywords:** magnetic polymer microspheres, chromium(VI), adsorption, kinetic, isotherms

## Abstract

Magnetic polymer microspheres (MPMs) using glycidylmethacrylate (GMA) as a functional monomer were synthesized in the presence of Fe_3_O_4_ nanoparticles via dispersion polymerization. After polymerization, the magnetic polymer microbeads were modified with ethylenediamine (EDA). The obtained ethylenediamine-functionalized magnetic microspheres (EDA-MPMs) were characterized by scanning electron microscope (SEM), X-ray diffraction (XRD), vibrating-sample magnetometer (VSM) and Fourier transform infrared (FT-IR) spectroscopy. Then the EDA-MPMs were applied as adsorbents for the removal of Cr(VI) from aqueous solution. Langmuir equation was appropriate to describe the experimental data. The maximum adsorption capacities obtained from the Langmuir model were 236.9, 242.1 and 253.2 mg/g at 298, 308 and 318 K, respectively. The Cr(VI) adsorption equilibrium was established within 120 min and the adsorption kinetics was compatibly described by the pseudo-second order equation. The thermodynamic parameters (Δ*G*°, Δ*H*°, Δ*S*°) of the sorption process revealed that the adsorption was spontaneous and was an endothermic process. The regeneration study demonstrated that the EDA-MPMs could be repeatedly utilized with no significant loss of adsorption efficiency.

## 1. Introduction

Heavy metal pollution is one of the most important environmental quality and human health problems today. Among the heavy metals, chromium is one of the most hazardous heavy metals, which is used extensively by several industries, including dyes, electroplating, textile and alloying [1,2]. Chromium exists in two oxidation states, Cr(VI) and Cr(III) [3]. The Cr(VI) form is 500 times more toxic to human health than the trivalent one [4]. Soluble Cr(VI) pollutes the soil and water, which would cause lung cancer [5]. Hence, it is necessary to remove Cr(VI) from wastewater to prevent the tremendous threat of Cr(VI) on ecosystem and public health. A large number of treatment methods in Cr(VI) removal have been reported, including adsorption [6,7], membrane separation [8,9], coagulation and precipitation [10,11], ion exchange [12,13] and solvent extraction [14]. Compared with other methods, adsorption is generally recognized as the most promising and widely used feasible technique due to its low cost, high efficiency, regeneration ability and friendly to environment [15,16]. A variety of adsorbents have been studied for Cr(VI) removal such as active carbon [17,18], metal oxide nanoparticles [19,20], synthesized polymer beads [4,21] and agriculture waste [22,23], *etc*. Bansal *et al.* reported that the adsorption capacity of rice husk carbon for the removal of Cr(VI) was evaluated to be 48.31 mg/g [24]. The adsorption of hexavalent chromium onto surfactant modified montmorillonites [25] and zeolitic material [26] was studied, with the maximum adsorption capacities of 18.05 and 75.8 mg/g, respectively. Metal oxide nanoparticles such as ZnO were employed as adsorbents for Cr(VI) from aqueous solution, and the maximum adsorption capacities were 9.38 mg/g [19]. These studies shows that these materials have inferior performance for the removal of Cr(VI) from aqueous solutions. Thus, it is very significant to explore an adsorbent with high adsorption capacity.

Synthesized polymer materials have been extensively used as adsorbents for removal of toxic heavy metal ions in recent years since the surface properties of the adsorbents can modified by the by the available functional groups to promote their adsorption ability. Several polymeric adsorbents have been used for removal of Cr(VI) such as glycine doped polypyrrole [27], 1,2-ethylenediamine-aminated macroporous polystyrene particles [28], magnetic poly-(MA-DVB) microspheres [29] and polyethyleneglycolmethacrylate-co-vinylimidazole microspheres [30]. Glycidyl methacrylate (GMA) has a vinyl group and an epoxide group, which are possible for various material preparations through radical polymerization and ring-opening reactions. Hwang *et al.* reported the preparation of poly(GMA-co-PEGDA) microbeads modified with iminodiacetic acid and their indium adsorption properties [31]. The epoxide group of GMA has a high reactivity with amine compounds, and amino-functionalized adsorbents showed an outstanding ability in the removal of Cr(VI) from wastewater [32]. Since amino groups are easily protonated under acidic conditions, the Cr(VI) can be adsorbed onto the adsorbent by electrostatic interaction and ion exchange.

To improve the adsorption efficiency, adsorbents are usually made into a very small size. But it would be very difficult to separate them from solution after accomplishing adsorption [33]. Recently, magnetic separation technique has attracted many researchers’ attentions because it makes the recovery of polymeric adsorbents from solution quite easy and simple under an external magnetic field [34]. Magnetic polymeric adsorbents are usually composed of the magnetic cores to ensure a strong magnetic responsibility and a polymeric shell to provide functional groups for various applications [35].

In the present work, Fe_3_O_4_ nanoparticles modified by Sodium dodecyl sulfate (SDS) and Polyethylene glycol (PEG) were synthesized by co-precipitation of Fe^2+^ and Fe^3+^ salts. Next, magnetic microbeads were prepared from glycidyl methacrylate and poly(ethylene glycol) diacrylate in the presence of Fe_3_O_4_ nanoparticles via dispersion polymerization. Then EDA-MPMs were grafted by ethylenediamine and were used to adsorb Cr(VI) ions in a batch system. The morphology and structure of the microbeads were confirmed by scanning electron microscope (SEM), vibrating-sample magnetometer (VSM), X-ray diffraction (XRD) and Fourier transform infrared (FT-IR) spectroscopy. The effects of several process parameters such as the initial concentration of Cr(VI) solution, initial pH value of Cr(VI) solution, contact time and reusability were investigated. In addition, the kinetic isotherm and thermodynamics about the adsorption of Cr(VI) on the m-poly(GMA-co-PEGDA)-EDA microbeads were also studied.

## 2. Results and Discussion

### 2.1. Morphologies of the Particles

**Figure 1:**
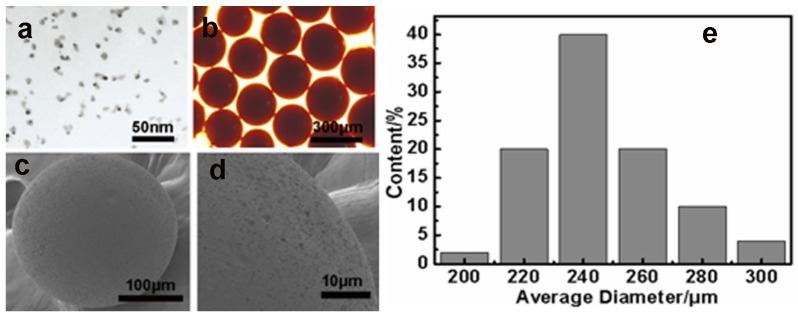
Electron and optical microscopy images of the products: (**a**) transmission electron microscope (TEM) image of Fe_3_O_4_ nanoparticles); (**b**) Optical microscopy of ethylenediamine-functionalized magnetic microspheres (EDA-MPMs); (**c**,**d**) scanning electron microscope (SEM) images of EDA-MPMs; (**e**) The histograms of size distribution of EDA-MPMs.

The transmission electron microscope (TEM) image of Fe_3_O_4_ nanoparticles was shown in Figure 1a. It can be revealed that the average diameter of Fe_3_O_4_ was about 10 nm with excellent dispersion property. According to the SEM pictures in Figure 1b,e, we can see that the diameter of microspheres has a narrow size distribution, and the size ranges from 200 to 300 μm. It is clear that ethylenediamine-functionalized magnetic microspheres (EDA-MPMs) are good spherical shape from Figure 1b,c. In Figure 1d, it is obviously shown that the surface of the EDA-MPMs is primarily rough. Additionally, the surface area, pore volume and pore diameter of EDA-MPMs are 286 m^2^/g, 0.512 cm^3^/g and 6.8 nm by Brunauer-Emmett-Teller (BET) analyses, which have higher surface area than other adsorbents reported by other literature [28].

### 2.2. Magnetism Analysis

**Figure 2:**
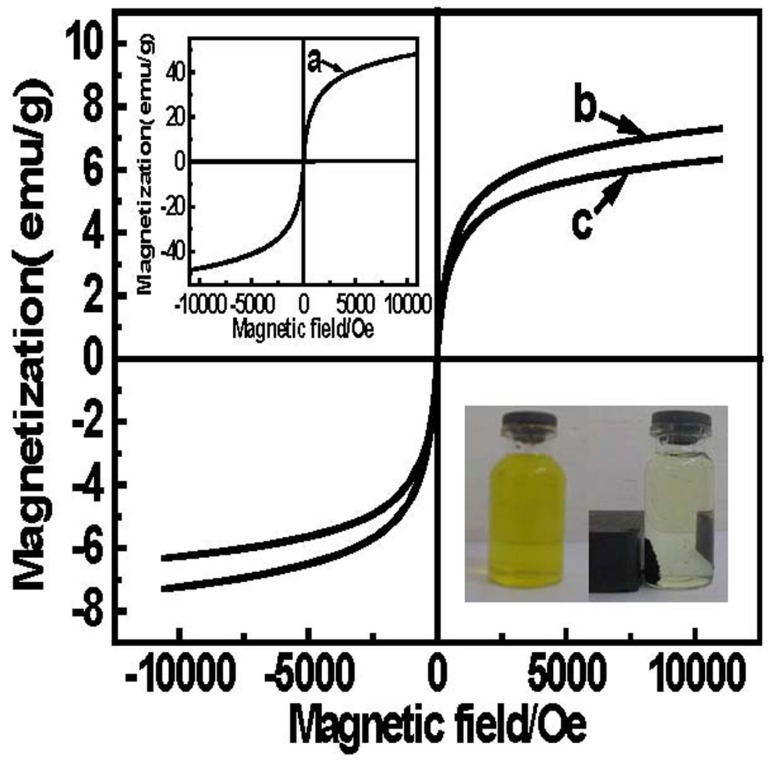
The magnetic hysteresis loop of magnetic nanoparticles (**a**); MPMs (**b**) and EDA-MPMs (**c**) at room temperature.

The value of saturated magnetization is an important parameter of magnetic materials, which reflects the ability of magnetic materials to respond to an external magnetic field. The data of coercivity and remanence demonstrates that magnetic microbeads exhibit superparamagnetism and the Fe_3_O_4_ nanoparticles remain in microspheres. From the curves in Figure 2a, the saturation magnetization of Fe_3_O_4_ nanoparticles was 45.5 emu/g. It is observed in Figure 2b,c that the saturated magnetization values of magnetic polymer microspheres (MPMs) and EDA-MPMs are 7.34 and 6.32 emu/g, respectively. Since Fe_3_O_4_ particles on the surface of the microbeads are converted to Fe_2_O_3_ during ring-opening reactions, saturated magnetization value between the two magnetic microbeads is different. With such saturation magnetization, EDA-MPMs could be easily and quickly separated from the aqueous solution by an external magnetic field.

### 2.3. X-ray Diffraction

**Figure 3:**
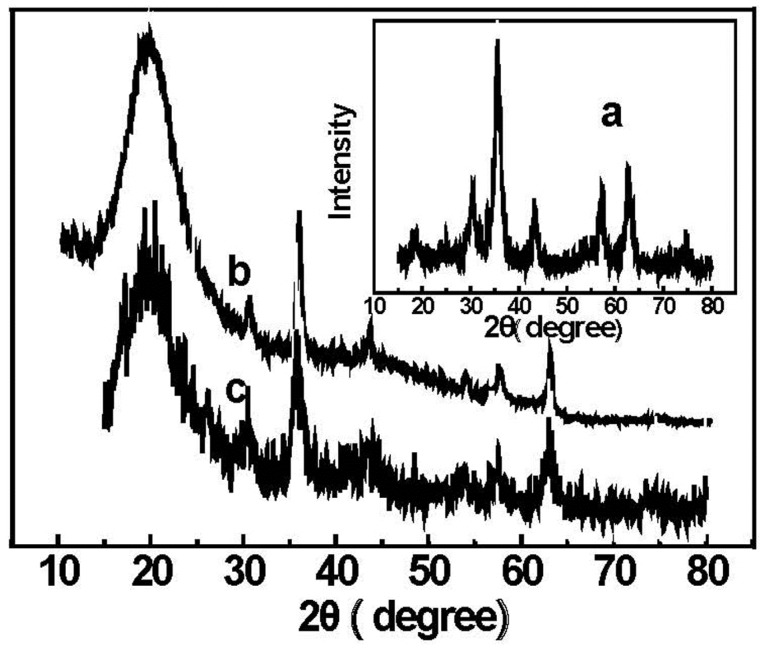
XRD spectrum of (**a**) Fe_3_O_4_ nanoparticles; (**b**) MPMs and (**c**) EDA-MPMs.

X-ray powder diffraction patterns of the bare Fe_3_O_4_ particles (a) MPMs (b) and EDA-MPMs (c) are illustrated in Figure 3, respectively. In Figure 3a, the XRD pattern of Fe_3_O_4_ particles shows main peaks at 2θ of 31°, 36°, 43°, 53°, 57° and 63°, corresponding to (220), (311), (400), (422), (511) and (440) Bragg reflection, respectively. It can be seen that the crystalline pattern coincides very well with the standard pattern of Fe_3_O_4_. Such a crystal system is important for the magnetic properties to be maintained. It can be found in Figure 3b that the polymer encapsulation can not change the typical spectra of Fe_3_O_4_ nanoparticles. The diffraction peaks at 31°, 36°, 43°, 53°, 57° and 63° for the EDA-MPMs are consistent with that of Fe_3_O_4_ nanoparticles. From the XRD pattern of EDA-MPMs (c), it was observed that the intensity of diffraction peaks was weakened but did not disappear. Additionally, the new diffraction peaks at 2θ = 25°, 33°, 39°, 41°, 49°, 54° and 73° suggested the presence of Fe_2_O_3_.

### 2.4. Fourier Transform Infrared (FT-IR) Analysis

**Figure 4:**
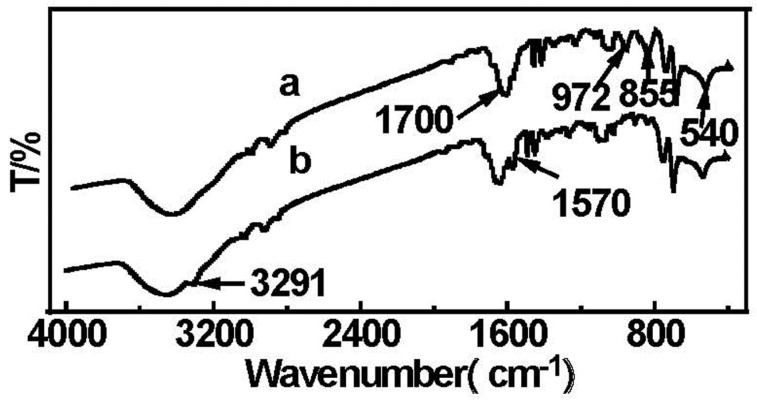
FT-IR spectra of MPMs (**a**) and EDA-MPMs (**b**).

Figure 4 shows the FT-IR spectra of MPMs and EDA-MPMs. The typical peak of Fe-O bond appeared at 540 cm^−1^ for the spectra in Figure 4a,b, which proved that the Fe_3_O_4_ nanoparticles remained in the polymer. As shown in Figure 4a, the strong band at 1700 cm^−1^ corresponded to C=O vibrations, and the bands at 855 and 972 cm^−1^ were attributed to the epoxy groups on the MPMs. After ring opening reaction with EDA in Figure 3b, the bands at 855 and 972 cm^−1^ disappeared, and new bands appeared at 1570 and 3291 cm^−1^, which were corresponding to the N–H vibrations in the EDA. These results clearly indicated that the ester groups have successfully reacted with EDA on the MPMs. In addition, the higher nitrogen amount (7.01%) found in EDA-MPMs confirmed that a polymer shell had been grafted with amino groups by elemental analysis.

### 2.5. Adsorption

#### 2.5.1. Effect of pH on Cr(VI) Adsorption and Sorption Mechanism

**Figure 5:**
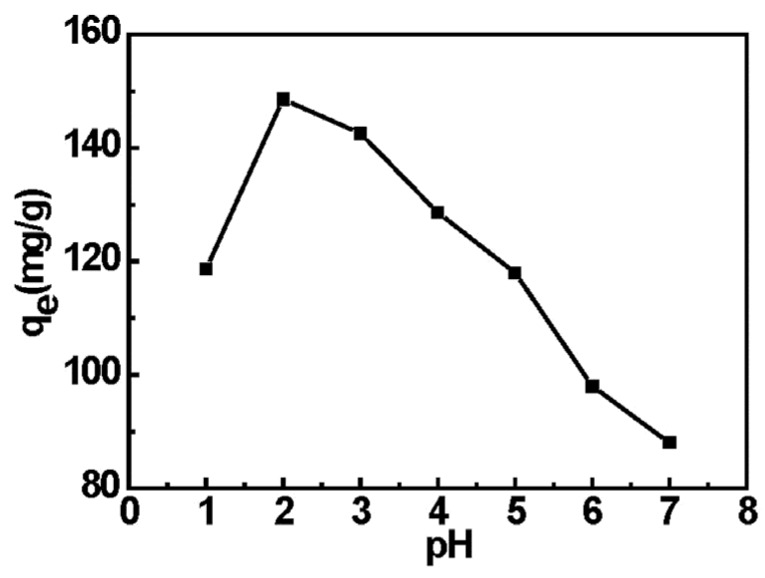
Effect of the pH on the Cr(VI) adsorption onto EDA-MPMs.

The pH of the Cr(VI) solution plays an important role in the adsorption process. It has an effect on the conversion of chromium species in the solution as well as the surface charge and the protonation degree of functional groups on the active sites of the adsorbent. There are five main forms of Cr(VI) in the aqueous solution, including Cr_2_O_7_^2−^, HCr_2_O_7_^−^, CrO_4_^2−^, HCrO_4_^−^ and H_2_Cr_2_O_4_n [36]. The stability of these forms is dependent on the pH of the system. When pH < 1, the chromium ions exist in the form of H_2_CrO_4_, while HCrO_4_^−^ and Cr_2_O_7_^2−^ are predominant at pH 2.0–6.0 and CrO_4_^2−^ is the dominant component of Cr(VI) above pH 6.0 [32]. In this study, the effect of initial pH value on Cr(VI) adsorption amount on EDA-MPMs was illustrated in Figure 5 at different pH values ranging from 1.0 to 8.0. The results showed that the maximum adsorption capacity on EDA-MPMs was at pH 2.0. At pH 2.0, the amino groups of the adsorbent was the protonated cations (–NH_3_^+^) which resulted in a stronger attraction for a negatively charged ion in the solution. The negatively charged chromium species (HCrO_4_^−^ and Cr_2_O_4_^2−^) were the predominant species, which were easily attracted through electrostatic attraction to the positively charged surface of the EDA-MPMs. Therefore, the maximum adsorption capacity was achieved at pH 2.0 (148 mg/g) in the initial Cr(VI) concentration of 300 mg/L, which was consistent with the previous study [28,37]. But in acidic medium (pH < 2), the neutral H_2_CrO_4_ was the main component, which brought about the decline of electrostatic attraction between adsorbent and Cr(VI). Hence, the adsorption capacity decreased as the pH decreased from pH 2.0 to 1.0 due to the involvement of less number of HCrO_4_^−^. At higher pH, the adsorption capacity decreased due to the competition of both the anions (CrO_4_^2−^ and OH^−^) to be adsorbed on the surface of the adsorbent of which OH^−^ tend to repulse the metal anions (CrO_4_^2−^). Therefore, the optimal pH value of 2.0 was selected for the subsequent experiments.

The adsorption process of the Cr(VI) can be explained in terms of the electrostatic interactions between the positively charged protonated functional groups (–NH_3_^+^) on EDA-MPMs surface and the negative charge chromium species (HCrO_4_^−^ and Cr_2_O_4_^2−^). The amino groups of EDA-MPMs were protonated under acidic conditions according to the following reaction.

R–NH_2_ + H^+^ ↔ R–NH_3_^+^ (1)

The chromium adsorption reactions onto EDA-MPMs are shown in Equations (2) and (3).

HCrO_4_^−^ + R–NH_3_^+^ ↔ HCrO_4_^−^ ··· ^+^NH_3_–R (2)

Cr_2_O_4_^2−^ + R–NH_3_^+^ ↔ CrO_4_^2−^ ··· ^+^NH_3_–R (3)

#### 2.5.2. Effect of Contact on MPMs and EDA-MPMs

**Figure 6:**
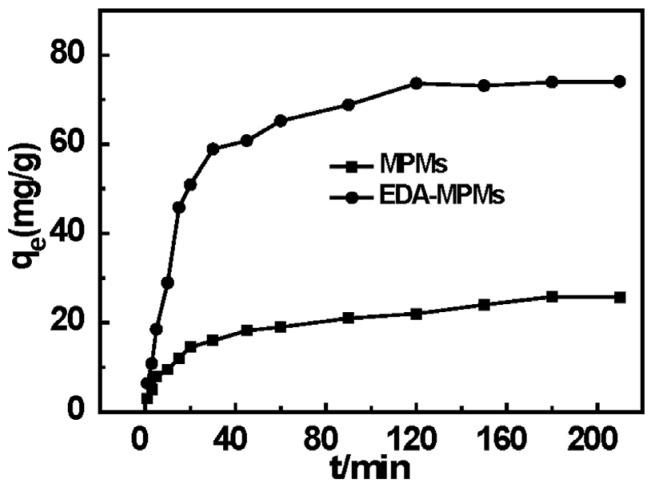
Effect of sorption time on the Cr(VI) adsorption onto MPMs and EDA-MPMs at different concentrations.

The effect of sorption time on the adsorption of Cr(VI) was also investigated from 1 to 210 min under pH = 2, at 298 K and initial concentration of 200 mg/L. As can be clearly seen from Figure 6, the Cr(VI) adsorbability on the magnetic microbeads increased nonlinearly with the increasing time. For MPMs, the sorption of Cr(VI) ion onto the MPMs rose slowly and reached equilibrium after 180 min. For EDA-MPMs, the initial rate was rapid and about 80% uptake of Cr(VI) was achieved during the first 30 min and the remaining amount removal occurred in the following 90 min. In comparison with MPMs, the initial rapid step of Cr(VI) sorption on EDA-MPMs may be attributed to electrostatic attraction. At low pH, the amino groups of the adsorbent were the protonated cations (–NH_3_^+^ or –NH_2_^−^–) and were bared on the surface of EDA-MPMs which emerged a facilely immediate interaction between Cr(VI) ions and the active. The Cr(VI) sorption capacity of EDA-MPMs will be primarily studied in the following substance.

#### 2.5.3. Adsorption Kinetics of the EDA-MPMs

In order to better illustrate the adsorption mechanism of EDA-MPMs for Cr(VI), adsorption kinetics is essential and important for evaluation of the adsorption behavior. The pseudo-first order and the pseudo-second order kinetic models are employed to evaluate the experimental data obtained from batch Cr(VI) removal experiments. The two kinetic models equations are given as follows:

Pseudo-first-order Equation [38]:

(4) $$\ln(q_{e}-q_{t})=\ln q_{e}-k_{1}t$$

Pseudo-second-order Equation [39]:

(5) $$\frac{t}{q_{t}}=\frac{1}{k_{2}q_{e}^{2}}+\frac{t}{q_{e}}$$

where *q_e_* and *q_t_* are the amounts of solute adsorbed at equilibrium and at time *t* (min), respectively; *k*_1_ (1/min) and *k*_2_ (g·mg^−1^·min^−1^) are the kinetic rate constants for the pseudo-first-order and the pseudo-second-order models, respectively.

**Figure 7:**
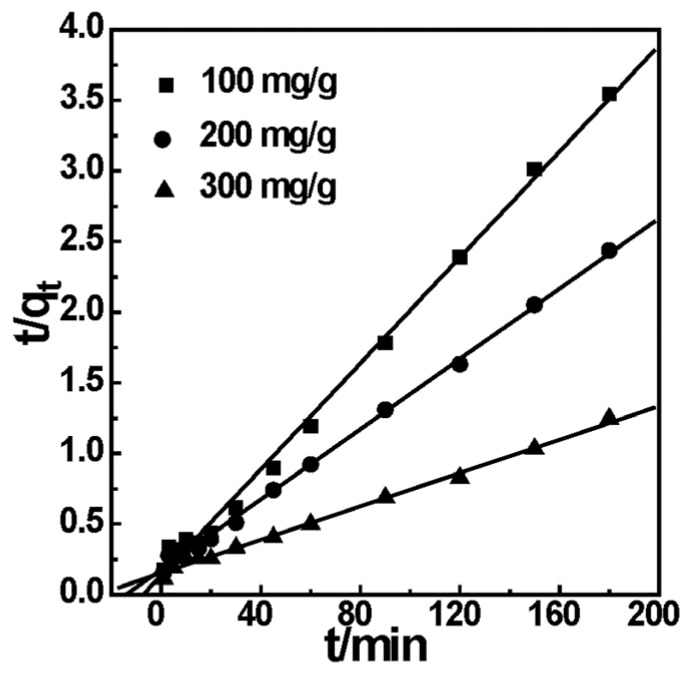
Linear fit of experimental data using pseudo-second-order kinetic model.

All the corresponding parameters obtained from the linear form of pseudo-first-order and pseudo-second-order are listed in Table 1 and Table S1. The fitting curves obtained from the linear plots of *t*/*q_t_ versus* *t* are plotted in Figure 7 and Figure S1. It can be seen that the linear correlation coefficients (*R*^2^) for pseudo-second-order kinetic model are all over 0.9990. Therefore, the pseudo-second-order kinetic model is able to describe properly the kinetic behavior of Cr(VI) on the EDA-MPMs. Furthermore, the *k*_2_ value decreased from 25.539 × 10^−4^ to 2.295 × 10^−4^ g·mg^−1^·min^−1^ with an increase in the initial Cr(VI) concentration. The theoretical simulated curve fitted quite well with the experimental data in Figure 6. More importantly, the *q_e_* values calculated by pseudo-second-order model were in agreement with experimental values. Analogous results had also been reported by many researchers for different systems [40,41].

**Table 1:** Parameters of kinetic models for Cr(VI) adsorption onto the ethylenediamine-functionalized magnetic microspheres (EDA-MPMs).

*C*_0_ (mg/g)	*q_e, exp _^a^* (mg/g)	Pseudo-First Order Model			Pseudo-Second Order Model		
		*q_e,cal_* (mg/g)	*k*_1_	*R*^2^	*q_e,cal _^b^* (mg/g)	*k*_2_ (g·mg^−1^·min^−1^) × 10^−4^	*R*^2^
100	50.5	14.206	0.0319	0.803	53.333	25.539	0.998
200	73.9	56.179	0.0321	0.968	80.451	8.718	0.999
300	145.2	141.206	0.0303	0.985	169.205	2.295	0.997

*^a^ q_e,exp_*: the experimental *q_e_* values; *^b^ q_e,cal_*: the calculated *q_e_* values.

#### 2.5.4. Adsorption Isotherm

Adsorption isotherm is one of the most important considerations in the application of adsorbents. Langmuir and Freundlich equations were fitted to the experiment data to investigate the isotherm type. The assumption of the Langmuir equation describes that the adsorption is a homogeneous surface by monolayer adsorption without interaction between adsorbed sites. The Freundlich isotherm equation is based on the assumption that the adsorption of EDA-MPMs occurs on a heterogeneous surface by multilayer adsorption.

Langmuir Equation [42]:

(6) $$\frac{c_{e}}{q_{e}}=\frac{c_{e}}{q_{m}}+\frac{1}{q_{m}b}$$

Freundlich Equation [43]:

(7) $$\ln q_{e}=\ln K_{F}+\frac{1}{n}\ln c_{e}$$

where *c_e_* (mg/L) is the remaining Cr(VI) concentration in solution; *q_e_* (mg/g) is the equilibrium amount of magnetic microbeads absorption; While *q_m_* (mg/g) represent the maximum adsorption amount of Cr(VI) and *b* (L/mg) is the Langmuir adsorption equilibrium constant. *K_F_* (L/g) and n are the Freundlich constants, which indicate the adsorption capacity and intensity, respectively.

**Figure 8:**
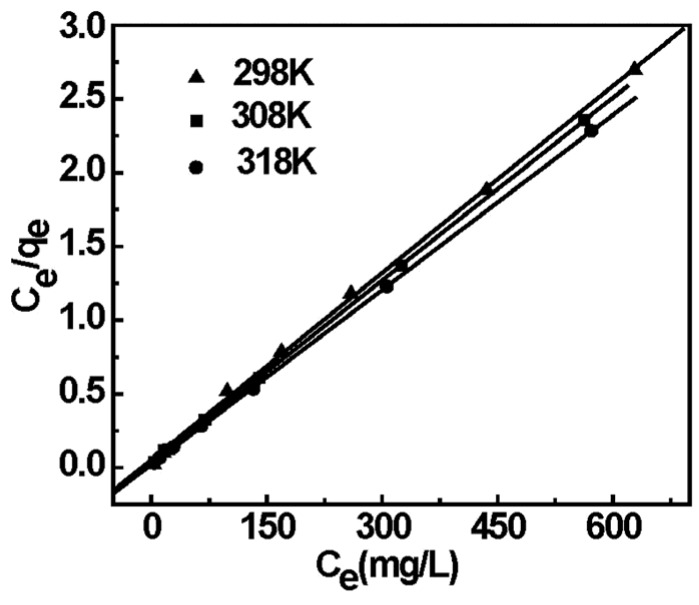
Linear fit of experimental data using Langmuir adsorption isotherm model.

The isotherm data has been linearized using Langmuir and Freundlich isotherm and the constants involved with the models and the correlation coefficients are listed in Table 2 and Table S2. In addition, the isotherm data is plotted between *c_e_*/*q_e_ versus c_e_*, which is shown in Figure 8 and Figure S2. Compared with two isotherm models, the Langmuir model describes the experimental data better than the Freundlich isotherm due to the higher correlation coefficients (*R*^2^ > 0.999). Based on the assumptions of the Langmuir model, the adsorption process belongs to monolayer adsorption. The Langmuir constant *q_m_* for Cr(VI) by EDA-MPMs is close to the experimental data. The maximum adsorption capacities at 25–45 °C are 236–253 mg/g. Most importantly, the maximum adsorption capacity of EDA-MPMs is higher than those of other adsorbents. The values of some of these Cr(VI) adsorbents were given in Table 3. The high capacity indicated that the EDA-MPMs have a potential application in the removal of Cr(VI) from wastewater.

**Table 2:** Isotherm constants for the adsorption of Cr(VI) onto the EDA-MPMs.

Temperature (K)	Langmuir Equation			Freundich Equation		
	*q_max_* (mg/g)	*b* (L/mg)	*R*^2^	*K_F_*	*n*	*R*^2^
298 K	236.9	0.0752	0.999	110.132	7.512	0.901
308 K	242.1	0.127	0.999	84.612	5.011	0.944
318 K	253.2	0.183	0.999	105.314	6.288	0.922

**Table 3:** Comparison of Cr(VI) adsorption capacity in other adsorbents.

Adsorbent	pH	Adsorption Capacity (mg/g)	References
MSCGE	2.0	171.5	[32]
Magnetic poly(MA-DVB) microspheres	3	231.8	[44]
Aminofunctionalized titanate nanotubes	5.4	153.85	[45]
Polyethylenimine-modified magnetic nanoparticle	2–3	83.33	[46]
EDA-MPMs	2	253	This Study

#### 2.5.5. Adsorption Thermodynamics

**Figure 9:**
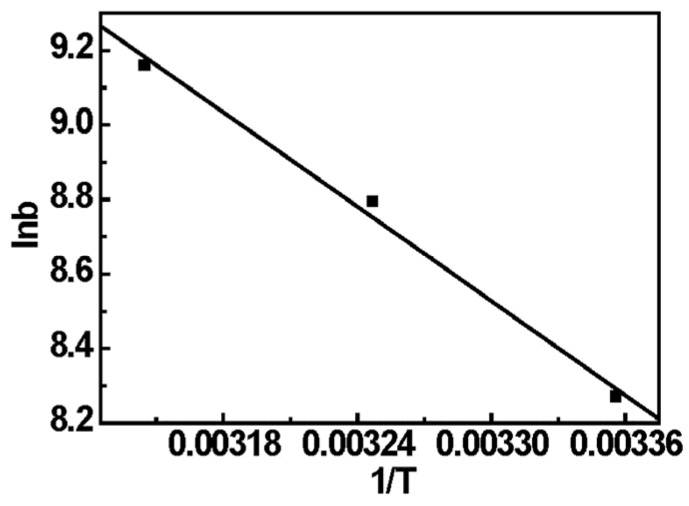
Linear plots of ln*b versus* 1/*T* for the adsorption of Cr(VI) onto EDA-MPMs.

The thermodynamic parameters, including the Gibbs free energy change (Δ*G*°), enthalpy change (Δ*H*°), and entropy change (Δ*S*°), were calculated from the slopes and intercept of plots of Van’t Hoff plot (ln*b versus* 1/*T*) as shown in Figure 9 by using the following equations:

(8) $$\ln b=-\frac{\Delta H^{o}}{RT}+\frac{\Delta S^{\text{o}}}{R}$$

The Gibbs free energy change (Δ*G°*) was calculated from the following equations:

(9) $$\Delta G^{\text{o}}=\Delta H-T\Delta S^{\text{o}}$$

where *b* represents the thermodynamic equilibrium constant, which can be obtained from Langmuir isotherms model at different temperature; *R* is the universal gas constant (8.314 J·mol^−1^·K^−1^); and *T* is absolute solution temperature (K).

The values of Δ*H*°, Δ*S*° and Δ*G*° for Cr(VI) adsorption on EDA-MPMs are presented in Table 4. The negative value of the Δ*G*° confirms that the adsorption of Cr(VI) onto EDA-MPMs is a spontaneous process. The Gibbs energy of the interactions demonstrates that the processes are favorable for the Cr(VI)···(–NH_2_) electrostatic interaction. The positive value of Δ*H*° indicates that the adsorption process is an endothermic process. A higher temperature is favourable to the adsorption, which may explain the increase of *q_m_* with the raise of temperature. The positive value of Δ*S*° reflects an increase in the randomness at the solid/solution interface during the adsorption process of Cr(VI) on the EDA-MCMs.

**Table 4:** Thermodynamic parameters for Cr(VI) adsorption onto EDA-MPMs.

Temperature (K)	Δ*G*° (kJ/mol)	Δ*H*° (kJ/mol)	Δ*S*° (J/mol/K)	*R*^2^
298	−20.492	35.085	186.69	0.996
308	−22.522			
318	−24.219			

### 2.6. Desorption and Reusability Study

**Figure 10:**
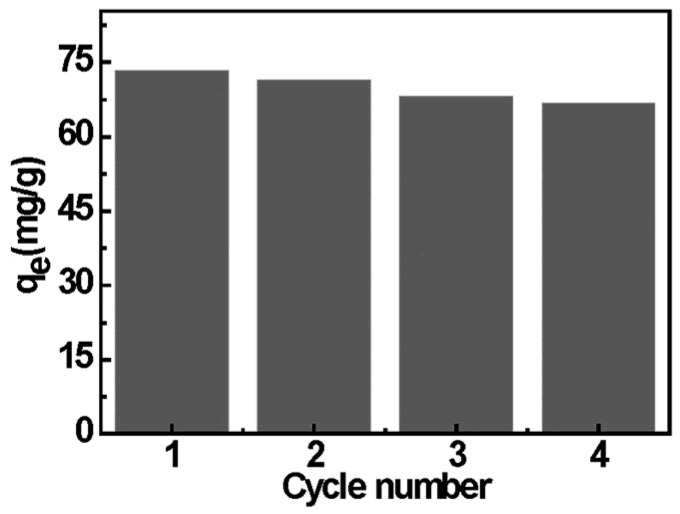
Adsorption/desorption cycles of Cr(VI) ions onto EDA-MPMs.

For the potential application of an adsorbent in wastewater treatment plant, the regeneration for reuse is very important. In order to investigate the reuse value of EDA-MPMs, adsorption-desorption process was repeated for four times. As can be shown from Figure 10, the relationship between the times for reuse and the adsorption efficiency of EDA-MPMs for Cr(VI) was illustrated. It can be seen that the adsorption capacity of the magnetic microbeads for Cr(VI) could still be maintained at over 90% level after the consecutive three-time adsorption-desorption processes. This suggests that the EDA-MPMs possess the potential of regeneration and reuse.

## 3. Experimental Section

### 3.1. Materials

Glycidyl methacrylate (GMA) and poly(ethylene glycol) diacrylate (PEGDA, *M_w_* = 258) were obtained from Ai Keda Chemical Technology Co., Ltd. (Chengdu, China) and were purified under reduced pressure. Benzoyl peroxide (BPO, 75%, used as an initiator) and Polyethylene glycol (PEG6000, used as a stabilizer) were purchased from the Sinopharm Chemical Reagents Co., Ltd. (Beijing, China). Ethylenediamine (EDA), ferric chloride hexahydrate (FeCl_3_·6H_2_O), ferrous chloride tetrahydrate (FeCl_2_·4H_2_O), potassium dichromate (K_2_Cr_2_O_7_), and *N*,*N*-dimethylformamide (DMF) were purchased from the Tianjin Kemiou Chemical Reagent Co. (Tianjin, China). All reagents above were of analytical grade.

### 3.2. Synthesis of the Adsorbent

**Scheme 1:**
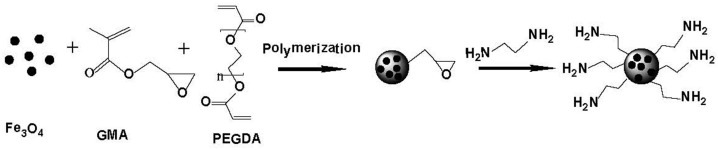
A schematic mechanism for synthesis of EDA-MPMs.

The synthesis route of EDA-MPMs is shown in Scheme 1.

#### 3.2.1. Preparation of Fe_3_O_4_ Nanoparticles Modified by SDS and PEG

Fe_3_O_4_ nanoparticles modified by SDS and PEG6000 were prepared by the co-precipitation method. 4 g of FeCl_3_·6H_2_O and 1.8 g of FeCl_2_·4H_2_O were dissolved in 100 mL distilled water and then were added into a 500 mL three-necked flask with a condenser, a nitrogen inlet and a stirrer. The system was heated up to the temperature of 55 °C and kept for 0.5 h. Next, 30 mL of 5 mol/L NaOH was quickly added into the reactive system. After reaction for 10 min, the 30 mL solution of 1 g PEG6000 and 0.5 g SDS was added dropwise into the system. The system was maintained at 55 °C and was stirred for 3 h. The products were washed by distilled water for several times until pH reached to neutral and stored.

#### 3.2.2. Synthesis of Magnetic Polymer Microspheres

The magnetic polymer microspheres were prepared by dispersion polymerization method. The mixture of 3 g PEG and 10 g magnetic fluid solution was added to 100 mL ethanol/water solution (volume ratio = 1:1). Then, the aqueous phase was moved to a 250 mL three-necked flask with a condenser, a nitrogen inlet and a mechanical stirrer. The polymerization system was then heated up to the temperature of 75 °C and was stirred at a rate of 400 rpm for 30 min under nitrogen atmosphere. Next, an oil phase mixture containing 2.5 g BPO, 5.6 mL PEGDA and 10.8 mL GMA was added into the flask by dropwise addition while keeping stirring (400 rpm) at 75 °C for 8 h. The final magnetic polymer microbeads were washed and stored for further applications.

#### 3.2.3. Ring-Opening Reaction of the Ester Groups

Two grams of the MPMs were dispersed in a mixture composed of 50 mL of EDA and 50 mL of *N*,*N*-dimethylformamide (DMF). Next, this solution was then kept at 80 °C for 12 h under stirring. The resulting product was washed several times with deionized water and ethanol, and then dried.

### 3.3. Characterization

The Fe_3_O_4_ nanoparticles were observed in a transmission electron microscope (TEM, JEOL JEM-3010, Tokyo, Japan). A scanning electron microscope (SEM, XL-30, Philips Corp., Eindhoven, Holland) was used to examined the morphology of EDA-MPMs. Optical microscope (Olympus, Japan) was used to determine the diameter and diameter distribution of the MPMs. The FT-IR spectra of MPMs and the EDA-MPMs were measured using Fourier transform infrared (FT-IR) spectroscopy (IR Prestige-21, Shimadzu, Tokyo, Japan) in the range from 4000 to 500 cm^−1^. The vibrating sample magnetometer (VSM, LakeShore 7407, Westerville, OH, USA) was applied to investigate the magnetic samples at room temperature. The X-ray diffraction (Model D/Max2500PC Rigaku, Tokyo, Japan) patterns were taken from 15° to 85° (2θ value) using Cu Kα radiation. The elemental analysis of MPMs and EDA-MPMs was performed with a Vario EL Cube elemental analyzer (Elementar Analysen Systeme GmbH Vario E1, Hanau, Germany)

### 3.4. Batch Adsorption Experiments

All the adsorption experiments were carried out on a rotary shaker at 150 rpm. Fixed dose (0.05 g) of adsorbent was added into 50 mL of a solution of known Cr(VI) concentration in 250 mL flasks, and then the flasks were agitated to reach the adsorption equilibrium. Then the residual concentration of Cr(VI) was analyzed inductively coupled plasma (ICP) spectrophotometry (PE Optima 8000, Waltham, MA, USA). To investigate the effect of pH, 50 mL of 300 mg/L Cr(VI) solutions with various initial pH at 1–8 was carried out at 298 K. The pH value was adjusted by 1 mol/L NaOH or 1 mol/L HCl. Effects of sorption time and sorption kinetics experiment were investigated at an initial pH of 2.0 by measuring the adsorption amount at different time intervals. 0.05 g EDA-MPMs was added to 50 mL of Cr(VI) aqueous solutions with concentration (100, 200 and 300 mg/L) at different temperature.

Adsorption isotherm was measured by contacting 0.05 g EDA-MPMs microbeads with 50 mL of Cr(VI) aqueous solutions at different initial concentration (100, 150, 200, 300, 400, 600, 800 and 1000 mg/L) shaking for 8 h. The experiments were performed at three different temperatures (298, 308 and 318 K). The equilibrium adsorption capacity (*q_e_*) was calculated by Equation (1):

(10) $$q_{e}=\frac{(c_{0}-c_{e})V}{m}$$

where *c*_0_ and *c_e_* represent the initial and equilibrium Cr(VI) concentrations in solution (mg/L), respectively; *V* is the volume of solution (L) and *m* is the amount of adsorbent (g).

### 3.5. Desorption Experiments

The adsorption-desorption cycles were repeated consecutively four times to determine the reusability of sorbents. For adsorption experiments, 0.05 g of EDA-MPMs was loaded with Cr(VI) ions using 50 mL (200 mg/L) solution at 298 K, pH 2.0 and contact time of 8 h. The agitation rate was fixed at 150 rpm. After adsorption experiments, the Cr(VI)-loaded EDA-MPMs were separated from the solution by an external magnetic field; and then desorption of Cr(VI) was performed using 10 mL 0.1 mol/L NaOH. The final Cr(VI) concentration was determined by ICP-OES. After each cycle of adsorption–desorption, sorbent was washed with distilled water and used in the succeeding cycle.

## 4. Conclusions

High-capacity magnetic EDA-MPMs functionalized with amino groups were successfully synthesized and characterized by SEM, VSM, XRD and FT-IR. The EDA-MPMs were then employed for the removal of Cr(VI) from an aqueous solution. The results demonstrated that the initial solution’s pH had an apparent effect on the adsorption capacity and the optimum pH value for Cr(VI) adsorption was found at pH = 2. It was revealed that the adsorption process was a pseudo-second-order reaction and the equilibrium was established within 120 min. Langmuir isotherm model was in good agreement with the experimental data and the maximum adsorption capacity was evaluated to be 236.9 mg/g at 298 K, which was higher than those of other adsorbents reported in the literatures. Thermodynamic results indicated that the adsorption was a spontaneous and exothermic process. In addition, the reusability data during the consecutive four times adsorption-desorption processes illustrates that of EDA-MPMs is a promising absorbent for application. The above result indicates that the EDA-MPMs could be employed as a low cost adsorbent for the removal of both Cr(VI) from the aqueous solution.

## Supplementary Materials

Supplementary materials can be found at www.mdpi.com/1996-1944/8/12/5461/s1.
