# Supplementary material for: Removal of Chromium(VI) from Aqueous Solutions Using Fe3O4 Magnetic Polymer Microspheres Functionalized with Amino Groups
Source: Materials (Basel). 2015 Dec 3;8(12):8378–91. doi: 10.3390/ma8125461 (PMC5458844; doi:10.3390/ma8125461)
Supplement: Supplementary file 1 [file materials-08-05461-s001.pdf]

## Supplementary Materials

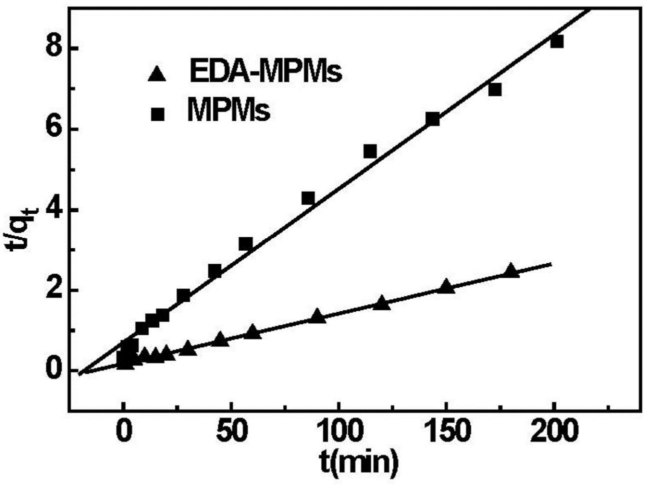


**Figure S1.** Linear fit of experimental data using pseudo-second-order kinetic model on EDA-MPMs and MPMs. (Volume, 100 mL; absorbent dose, 0.1 g; initial concentration, 200 mg/L; Temperature, 298 K).

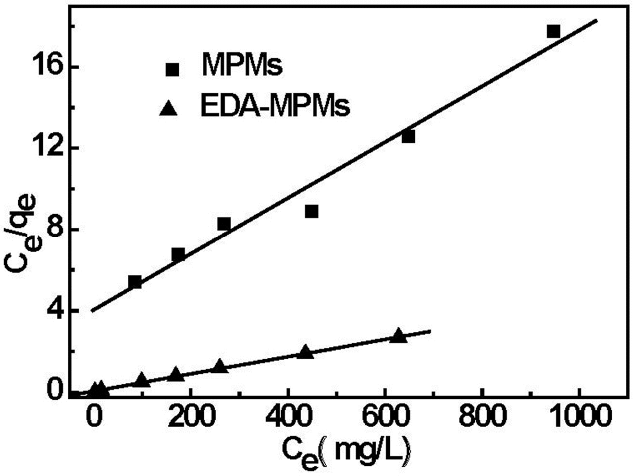


**Figure S2.** Linear fit of experimental data using Langmuir adsorption isotherm model on EDA-MPMs and MPMs. (Volume, 50 mL; absorbent dose, 0.05 g; pH value, 2.0; Temperature, 298 K).

**Table S1.** Parameters of kinetic models for Cr(VI) adsorption onto the EDA-MPMs. (Volume, 100 mL; absorbent dose, 0.1 g; initial concentration, 200 mg/L; Temperature, 298 K).

| Adsorbent | $q_{e,exp}$ (mg/g) | Pseudo-First Order Model | | | Pseudo-Second Order Model | | |
|---|---|---|---|---|---|---|---|
| | | $q_{e,cal}$ (mg/g) | $k_1$ | $R^2$ | $q_{e,cal}$ (mg/g) | $k_2$ (g/mg/min) × $10^{-4}$ | $R^2$ |
| MPMs | 26.4 | 21.624 | 0.0201 | 0.953 | 27.129 | 20.539 | 0.996 |
| EDA-MPMs | 73.9 | 56.179 | 0.0321 | 0.968 | 80.451 | 8.718 | 0.999 |

**Table S2.** Isotherm constants for the adsorption of Cr(VI) onto the EDA-MPMs at 298 K (Volume, 50 mL; absorbent dose 0.05 g; pH value, 2.0; Temperature, 298 K).

| $T$ (K) | Langmuir Equation | | | Freundich Equation | | |
|---|---|---|---|---|---|---|
| | $q_{max}$ (mg/g) | $b$ (L/mg) | $R^2$ | $K_F$ | $n$ | $R^2$ |
| MPMs | 66.88 | 0.0041 | 0.995 | 9.242 | 2.224 | 0.952 |
| EDA-MPMs | 236.9 | 0.0752 | 0.999 | 110.132 | 7.512 | 0.901 |
